# Flow Behavior of Chain and Star Polymers and Their Mixtures

**DOI:** 10.3390/polym10060599

**Published:** 2018-05-29

**Authors:** Deepika Srivastva, Arash Nikoubashman

**Affiliations:** Institute of Physics, Johannes Gutenberg University Mainz, Staudingerweg 7, 55128 Mainz, Germany; ddeepika@uni-mainz.de

**Keywords:** microfluidics, Poiseuille flow, simulations, polymers, stars, chains, separation

## Abstract

Star-shaped polymers show a continuous change of properties from flexible linear chains to soft colloids, as the number of arms is increased. To investigate the effect of macromolecular architecture on the flow properties, we employ computer simulations of single chain and star polymers as well as of their mixtures under Poiseuille flow. Hydrodynamic interactions are incorporated through the multi-particle collision dynamics (MPCD) technique, while a bead-spring model is used to describe the polymers. For the ultradilute systems at rest, the polymers are distributed uniformly in the slit channel, with a weak dependence on their number of arms. Once flow is applied, however, we find that the stars migrate much more strongly towards the channel center as the number of arms is increased. In the star-chain mixtures, we find a flow-induced separation between stars and chains, with the stars located in the channel center and the chains closer to the walls. In order to identify the origin of this flow-induced partitioning, we conduct additional simulations without hydrodynamic interactions, and find that the observed cross-stream migration originates from a combination of wall-induced hydrodynamic lift forces and viscoelastic effects. The results from our study give valuable insights for designing microfluidic devices for separating polymers based on their architecture.

## 1. Introduction

The ability to separate dispersed particles based on their properties, e.g., size, shape, or elasticity, is of immense importance for a large number of industrial and biological applications. For example, cell deformability is an important biomarker for diagnosing diseases: it has been demonstrated that cancer cells are significantly more deformable than healthy cells of the same tissue [1], and that the stiffness of red blood cells is highly affected by blood diseases such as sickle cell anemia [2]. In the past two decades, microfluidic devices have proven themselves as auspicious tools for the efficient separation of particles in solution [3,4,5,6,7,8,9,10,11]. The development of such devices is advantageous, as they can be operated continuously, thus allowing for high throughput and automation. Further, microfluidic devices are light and portable, require only low amounts of sample, and they can be manufactured cost-efficiently from polydimethylsiloxane (PDMS) [12].

To date, many different separation strategies have been developed, which can be roughly grouped into two categories [8]: active methods based on the application of (additional) external fields, and passive methods which exploit geometrical effects and/or (non-linear) hydrodynamic forces. In this work, we will focus on the latter approach, because it is label-free and therefore can be applied to a wide range of materials. Furthermore, non-linear hydrodynamic effects can be sustained or even amplified at high flow rates, guaranteeing high throughput.

Early studies have concentrated on the flow behavior of rigid particles in the μm to mm range, because these particle sizes can be resolved easily using optical microscopy. Here, it was found that the dispersed particles could be focused in the channel by exploiting inertial [13] or viscoelastic [14,15] flow effects. Due to the progressive miniaturization of microfluidic devices [16] and improvements of optical tracking techniques [17,18], it is now also possible to probe and manipulate particles in the nm regime. Here, it is of particular interest to study the behavior of macromolecules in microfluidic devices, for instance for genome sequencing [19,20] and polymer filtration [21,22,23,24]. In contrast to rigid particles, polymers are characterized by their many internal degrees of freedom and inherent flexibility, which can lead to strong deformation under flow [25,26]. A considerable amount of research has been conducted on the microrheology of fully flexible linear polymers, due to their rather simple architecture. Under Poiseuille flow, the chains move away from the channel center because of their position-dependent conformation and mobility (coiled in the center and stretched close to the walls), and are pushed away from the channel walls due to wall-induced hydrodynamic lift forces [27,28,29]. However, flexible linear polymers represent only a small subset of existing and conceivable macromolecules, and thus further research is necessary to elucidate the effects of, e.g., polymer architecture [21,22,23,24,30,31,32,33] and stiffness [34,35,36,37,38].

Star polymers, i.e., macromolecules consisting of *f* chains of length *p* attached to a common center, are particularly interesting, since they allow an almost continuous change from a flexible linear polymer to a spherical colloid with soft pair interactions [39,40,41,42,43]. (Hence, star polymers are often referred to as ultrasoft colloids). Owing to their macromolecular architecture, star polymers exhibit a strongly non-uniform core-corona morphology, which makes them promising candidates for various applications such as catalysis [44], photonics [45], and drug delivery [46]. Previous non-equilibrium studies of star polymers have focused on, e.g., the response to shear flow [30,31,47], the translocation through nanopores [48,49], and the sedimentation behavior under dilute conditions [50]. In this work, we are studying the behavior of single stars as well as of dilute mixtures of stars with different number of arms under Poiseuille flow. In particular, we are interested in the cross-stream migration of the macromolecules, and whether polymers can be separated spatially based on their architecture.

To establish a connection between the shape of star polymers and their flow properties, we carried out molecular dynamics (MD) simulations. Here, we modeled the polymers using a generic bead-spring description in order to focus on the general physical mechanisms, instead of replicating a specific polymer chemistry. Hydrodynamic interactions (HI) were incorporated using the multi-particle collision dynamics (MPCD) algorithm.

The rest of this manuscript is organized as follows. In Section 2, we present briefly the employed model and simulation method. In Section 3.1 we discuss first the flow behavior of single polymers at infinite dilution, and then discuss in Section 3.2 the results for polymer mixtures at finite concentration. Finally, we draw our conclusions and present our outlook in Section 4.

## 2. Model and Simulation Method

To study the dynamics of chain and star polymers under pressure driven flow, we combine standard MD simulations with the MPCD algorithm. This hybrid approach allows for taking into account long-ranged hydrodynamics in a physically accurate and computationally efficient way. We describe the dispersed macromolecules using a generic bead-spring model. In this representation, each star polymer consists of *f* linear chains with *p* beads each (often referred to as “arms”), which are attached to a common central particle (linear chains can be considered as star polymers with f=2). Thus, a polymer consists of N=fp+1 monomeric units in total. Each spherical bead has a diameter of σ, and the excluded volume interactions between the monomers are modeled through the purely repulsive Weeks-Chandler-Andersen (WCA) potential [51]

(1) $$\begin{array}{c} U_{\mathrm{WCA}}\left(r\right)=\left\{\begin{array}{cc} 4\varepsilon \left[{\left(\frac{\sigma }{r}\right)}^{12}-{\left(\frac{\sigma }{r}\right)}^{6}\right]+\varepsilon , & r\leq 2^{1/6}\sigma \\ 0, & r>2^{1/6}\sigma , \end{array}\right. \end{array}$$

where $r=|r_{j}-r_{i}|$ is the distance between particles *i* and *j*. The parameter ε controls the strength of the repulsion and has been set to $k_{B}T$.

The connection between consecutive monomers within a polymer is described by the finitely extensible nonlinear elastic (FENE) potential [52]

(2) $$U_{\mathrm{FENE}}\left(r\right)=-\frac{1}{2}kr^{2}\ln\left[1-\frac{r^{2}}{r_{0}^{2}}\right]$$

With the spring constant *k* and maximum bond length $r_{0}$. To prevent unphysical bond crossing, we have chosen $k=30\;\varepsilon /\sigma^{2}$ and $r_{0}=1.5\;\sigma$ [53]. With these parameters, the equilibrium bond length is b≈0.97σ.

**Figure 1:**
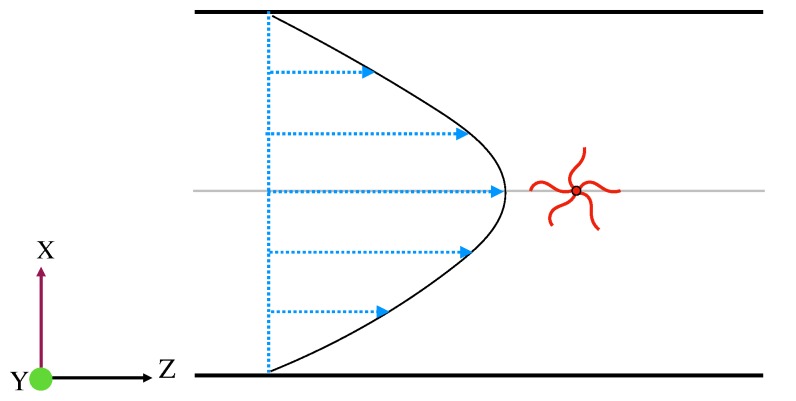
Schematic representation of the channel geometry and resulting flow profile.

All simulations are carried out in a slit-like channel with dimensions $L_{x}=40\;\sigma$ in the gradient direction, $L_{y}=40\;\sigma$ in the vorticity direction, and $L_{z}=50\;\sigma$ in the flow direction (see Figure 1 for a schematic representation of the channel geometry). Channel walls are modeled as infinitely extended smooth planes located at $x=\pm L_{x}/2$, which interact with the monomers through a purely repulsive potential along the wall normal [21].

In MPCD, solvent particles are modeled as ideal point particles with unit mass m=1, and their motion is governed by alternating streaming and collision steps [54,55]. During the streaming step, the solvent particles move ballistically for a time $\Delta t_{\mathrm{MPCD}}$. In the collision step, all solvent particles are first sorted into cubic cells of edge length *a*, which sets the length scale over which hydrodynamics are resolved [56]. Then, particles within the same cell exchange momentum through a stochastic collision, while conserving linear momentum on both the cellular and global level. In this work, we used an Andersen thermostat (MPCD-AT) collision scheme [57], which also acts as the thermostat in our simulations. The interaction between monomers and solvent particles is realized by including the monomers in the MPCD collision step. Cells were shifted before each collision by a random three-dimensional vector with components drawn uniformly on [−a/2,+a/2] to ensure Galilean invariance [58]. To enforce no-slip boundary conditions at the channel walls, we employed a bounce-back rule at the walls, and filled the cells that are intersected by the walls with virtual solvent particles [59]. Poiseuille flow was achieved by applying a body force *g* to all solvent particles [21,24,57,60,61].

The equation of motion for the dispersed solute particles is integrated using the standard velocity Verlet algorithm [62], with MD time step $\Delta t_{\mathrm{MD}}=2\times 10^{-3}\tau_{\mathrm{MD}}$ measured in the reduced unit of time $\tau_{\mathrm{MD}}=\sqrt{m\sigma^{2}/\left(k_{B}T\right)}$. The time step for the MPCD algorithm was set to $\Delta t_{\mathrm{MPCD}}=0.1$, i.e., a stochastic collision was performed every 50 MD steps. The cell size was set to a=σ and the number density of solvent particles was set to $\rho_{s}=5\;\sigma^{-3}$. The mass of the monomers was set to M=5 m. With these parameters, the pure MPCD solvent has a dynamic zero-shear viscosity of $\eta_{s}=3.71$ and a Schmidt number of Sc=8 [38], which is consistent with a liquid-like solvent [63]. Simulations were conducted up to $10^{5}\;\tau_{\mathrm{MD}}$, and we ensured that the systems reached a steady state before taking measurements. For every set of parameters, we conducted five independent runs to improve sampling and to calculate error bars. Due to the symmetry of the channel geometry, we consider only absolute displacements from the center line x=0 to improve sampling. Further, if not stated otherwise explicitly, we will use σ as our unit of length, $k_{B}T$ as our unit of energy, and $\tau_{\mathrm{MD}}$ as our unit of time. Simulations without hydrodynamics were performed using HOOMD-blue (version 2.2.4) [64,65,66].

## 3. Results and Discussion

### 3.1. Ultradiulte Conditions

In the first part of this work, we studied the flow behavior of single star polymers at infinite dilution for various arm numbers *f*. Here, we tuned the arm length *p* (and thus the total number of monomer *N*) so that, in an unconfined system, the polymers have roughly the same radius of gyration $R_{g}\approx 4.2$ at each value of *f*. In particular, we studied linear chains with N=40 monomers, and star polymers with f=18 (N=181) and f=30 (N=271).

**Figure 2:**
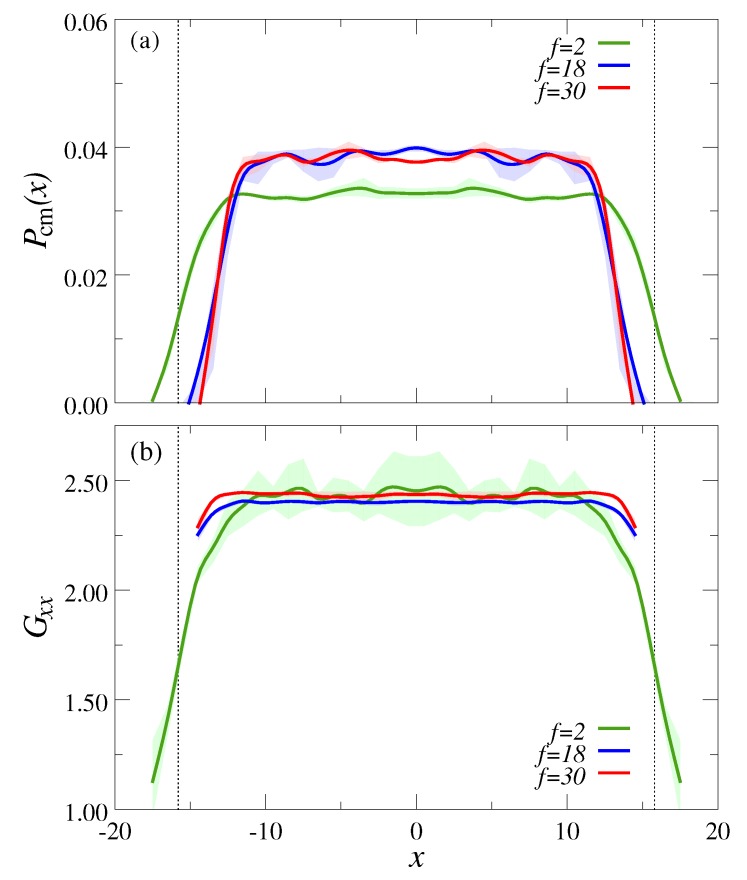
(**a**) Center of mass probability distribution normal to the channel walls, $P_{\mathrm{cm}}\left(x\right)$, for various arm numbers *f* at rest; (**b**) Component of the radius of gyration tensor normal to the walls ($G_{xx}$) as a function of center of mass distance *x*. In both panels, the shaded regions around the curves indicate our measurement uncertainty. The dotted vertical lines indicate the excluded regions of width $R_{g}$ near the channel walls.

Under quiescent conditions, the spatial distribution of the polymer center of mass (CM) between the channel walls, $P_{\mathrm{cm}}\left(x\right)$, is almost uniform, except for a narrow region of width ≈$R_{g}$ close to the channel walls (see Figure 2a). Note that the transition of $P_{\mathrm{cm}}\left(x\right)$ near the walls becomes significantly sharper with increasing *f*, since the interior of the polymers is packed more compactly with the constituent monomers, and thus it is more difficult to deform the macromolecule [43,67,68]. (For a completely hard colloid, $P_{\mathrm{cm}}\left(x\right)$ is a step function). To quantify the shape of the polymers, we computed the radius of gyration tensor

(3) $$\begin{array}{c} G_{\alpha \beta }^{2}=\frac{1}{N-1}\sum_{i}{\left(\Delta r_{i,\alpha }\Delta r_{i,\beta }\right)}^{2}, \end{array}$$

where $\Delta r_{i,\alpha }$ is the position of monomer *i* relative to the polymer CM, while α and β are the components along the Cartesian *x*, *y*, and *z* direction. The polymer radius of gyration is then given by $R_{g}^{2}\;=\;G_{xx}^{2}\;+\;G_{yy}^{2}\;+\;G_{zz}^{2}$. Figure 2b shows $G_{xx}$ between the channel walls, and it is clear that in the channel center $G_{xx}$ is independent of *f* and has the same value as in unconfined systems. When the polymer CM approaches the walls, however, $G_{xx}$ decreases drastically for the linear chains (f=2), whereas this effect is much weaker for the star polymers.

When a constant body force *g* is applied to the liquid along the *z*-direction, then a steady flow develops as a result of the balance between acceleration in the channel center and friction at the channel walls. Due to the low polymer concentration, the dispersion behaves essentially like a Newtonian liquid with shear viscosity $\eta =\eta_{s}$, and the resulting velocity profile is parabolic

(4) $$\begin{array}{c} v_{z}\left(x\right)=\frac{g}{2\nu }\left(\frac{L_{x}^{2}}{4}-x^{2}\right) \end{array}$$

where ν=η/ρ is the kinematic viscosity of the liquid. The velocity profile has its maximum $v_{\max}$ in the channel center (x=0) and becomes zero at the channel walls ($x=\pm L_{x}/2$). The parabolic shape of $v_{z}\left(x\right)$ leads to a locally varying shear rate $\dot{\gamma }\left(x\right)=dv_{z}\left(x\right)/dx=-gx/\nu$, which is maximum at the channel walls and vanishes in the channel center. We can estimate the average shear rate in the system by $\langle \dot{\gamma }\rangle \approx 2v_{\max}/L_{x}$. To facilitate the comparison with experiments and other simulations, we will express the flow strength in terms of the dimensionless particle Reynolds number, Rep=2vmaxRg/ν, which is the ratio between inertial and viscous forces acting on the dispersed polymer. Please note that this expression is somewhat approximative, as polymers can deform under flow and thus $R_{g}$ is not constant. Nevertheless, this quantity provides a reasonable measure for estimating the onset of inertial flow effects (Rep≳1) [24,27].

**Figure 3:**
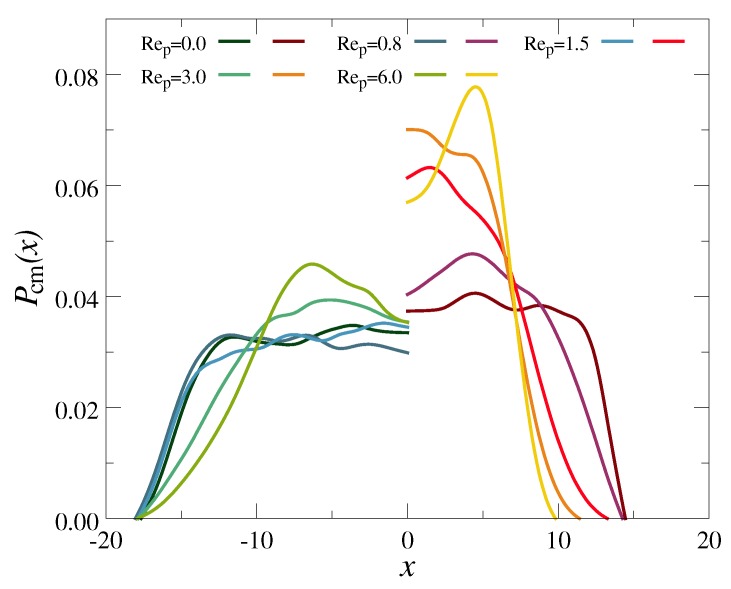
Center of mass probability distribution normal to the channel walls, $P_{\mathrm{cm}}\left(x\right)$, for a chain (**left**) and a star with f=30 arms (**right**) at various flow strengths Rep, as indicated.

**Figure 4:**
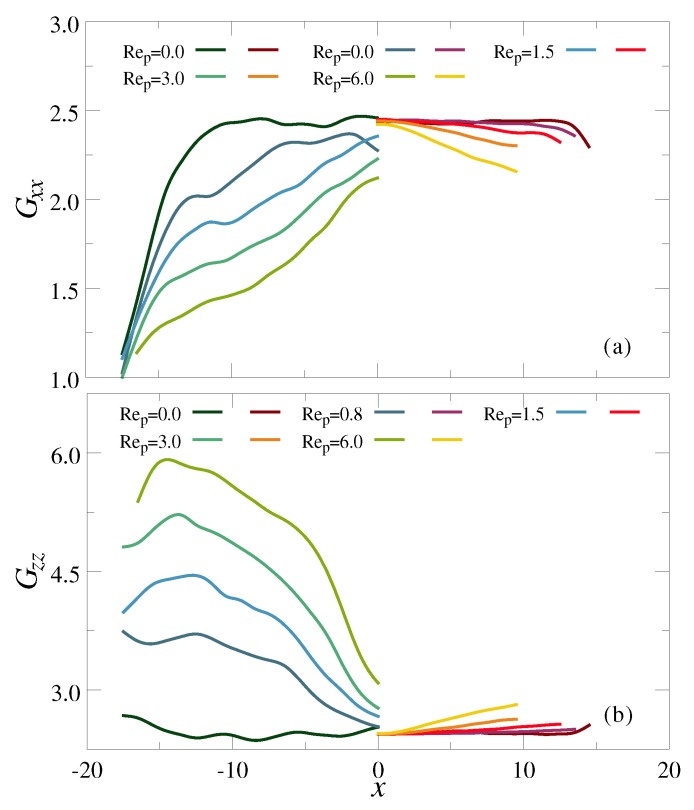
Components of the radius of gyration tensor along (**a**) the gradient and (**b**) the flow direction vs. the polymer CM position *x*. Data shown for a chain (**left**) and a star with f=30 arms (**right**) at various flow strengths Rep, as indicated.

In Figure 3, we have plotted how the CM distribution of the polymers, $P_{\mathrm{cm}}\left(x\right)$, changes under flow. As Rep is increased, both the chains (f=2) and stars (f=30) move away from the channel walls, due to the wall-induced asymmetry in the wake vorticity field of the dispersed polymers [27,28,36,37]. This cross-stream migration is significantly more pronounced for the star polymers compared to the linear ones, as the former contain almost seven times as many monomers (N=271 vs. N=40), and thus disturb the flow field to a greater extent. Further note that $P_{\mathrm{cm}}\left(x\right)$ develops a distinct dip near the channel center at the highest investigated flow rates, Rep=6, for the chains as well as the stars. This partial evacuation of the centerline originates from the nonuniform shear field $\dot{\gamma }\left(x\right)$, which leads to a position-dependent polymer deformation (see Figure 4 below) and a subsequent gradient in the chain mobility [27,28,36,37]. Star polymers with f=18 arms exhibit an intermediate behavior, and have been omitted from Figure 3 and Figure 4 for the sake of clarity.

Shear deformation of the dispersed polymers occurs typically when $\dot{\gamma }$ exceeds the inverse of the longest characteristic relaxation time, $\tau_{c}^{-1}$ (or, equivalently, when the Weissenberg number $\mathrm{Wi}\;\equiv \;\dot{\gamma }\tau_{c}≳$ 1) [25,30,31,32,38,69,70]. For linear chains in dilute solutions, $\tau_{c}$ is essentially given by the slowest Zimm relaxation mode, i.e., $\tau_{c}=\tau_{0}N^{3\nu }$ with $\tau_{0}=\eta_{s}b^{3}/\left(k_{B}T\right)$ and Flory exponent ν≈3/5 [25,71]. For star polymers, a similar calculation for the blob model leads to the expression $\tau_{c}=\tau_{0}p^{3\nu }f^{1-3\nu /2}$ [72], which has been verified through simulations [30] for the range of star sizes investigated here. One interesting result of these theoretical considerations is that a star relaxes of order $f^{-1/2}$ faster than a linear chain of the same overall $R_{g}$ [72]. For the polymers investigated in this work, we estimate $\tau_{c}=2600$ (linear chain), $\tau_{c}=285$ (f=18, p=10), and $\tau_{c}=250$ (f=30, p=9).

To investigate the flow-induced deformation of the polymers, we have plotted in Figure 4 the components of the radius of gyration tensor along the gradient and flow direction, $G_{xx}$ and $G_{zz}$, respectively, as a function of the polymer CM position between the walls *x*. (The size along the vorticity direction, $G_{yy}$, changed only marginally and therefore has been omitted for the sake of brevity.) Here, we can see that $G_{xx}$ in the channel center is almost independent of Rep, but then decreases gradually as the polymer CM approaches the high $\dot{\gamma }$ region close to the channel walls. Further, it is clear that, at a fixed CM distance, $G_{xx}$ drops with increasing flow strength since γ˙∝Rep. At the same time, the extension along the flow direction, $G_{zz}$, increases significantly both with distance to the centerline and flow strength. Comparing the $G_{\alpha \alpha }$ data for the chains and stars, it is clear that the flow-induced deformation is much more pronounced for the linear species. This finding can be rationalized by realizing that the characteristic relaxation time of the chain is approximately one order of magnitude slower than of the star (see discussion above). Further, even for Wi≫1, the compact structure of star polymers prevents full extension along the flow direction, as the arms would significantly overlap in such a scenario.

**Figure 5:**
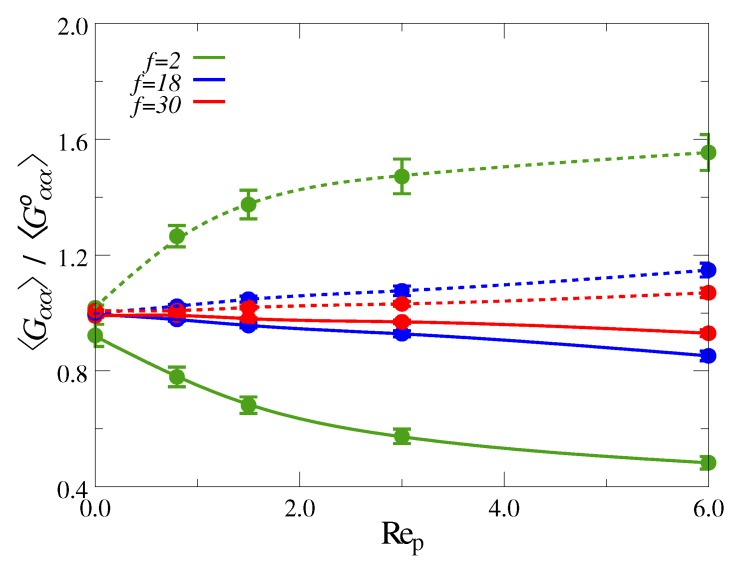
Components of the radius of gyration tensor averaged over the entire channel vs. flow strength Rep, normalized by the value at rest. Dashed lines show component in flow direction, $\langle G_{zz}\rangle$, and solid lines show component in gradient direction, $\langle G_{xx}\rangle$.

In Figure 5 we have plotted the *x* and *z* components of the radius of gyration tensor averaged over the entire channel, $\langle G_{xx}\rangle$ and $\langle G_{zz}\rangle$, respectively, as a function of Rep. Here, we can see that the average extension along the flow direction, $\langle G_{zz}\rangle$, is much more pronounced for the linear polymers compared to the star polymers, which exhibit only weak stretching. (The theoretical maximum of $G_{zz}$ for sheared linear polymers is on the order of half the chain contour length [35]). A similar trend can be observed for the average contraction in the gradient direction, $\langle G_{xx}\rangle$, which is significantly more expressed for the chains than for the stars. Thus, in this context, the star polymers resemble progressively rigid colloids as the number of arms *f* is increased. These findings can be explained by considering the average Weissenberg number $\langle \mathrm{Wi}\rangle \equiv \tau_{c}\langle \dot{\gamma }\rangle$, for which we find $\langle \mathrm{Wi}\rangle \approx 70$ for the chains and $\langle \mathrm{Wi}\rangle \approx 6.6$ for the stars (f=30) at Rep=6.

Based on previously established similarities between the elasticity of polymeric nanoparticles and deformable droplets at rest [43], it is tempting to also draw analogies for the flow-induced migration of the two species. Previous analytical models of droplets in the Stokes regime (Re≪1) predict that migration to the channel center should occur if the ratio of viscosities of the suspended phase and of the surrounding fluid is either smaller than 0.5 or larger than 10 [73,74]. In between these ratios, the droplets are predicted to move *away* from the centerline. However, recent simulations of deformable droplets in the inertial flow regime (Re≳1) have also found migration *towards* the centerline for viscosity ratios from unity to 13 [75]. Further, it was shown that these lift forces increase with increasing droplet deformability [75,76].

Applying those findings to the macromolecular particles studied here, one could then expect that the higher deformability of the linear chains should lead to stronger lift forces towards the channel center compared to the stars. This situation is, however, clearly *not* the case here as evidenced by the probability distribution $P_{\mathrm{cm}}\left(x\right)$ shown Figure 3. One possible explanation for this discrepancy could be that the solvent can (partially) flow through the polymers, whereas the droplets are completely impermeable. Further, the dynamics of polymers are governed by a hierarchy of relaxation times, originating from the many internal degrees of freedom.

We instead hypothesize that the cross-stream migration observed for the polymers stems from wall-induced hydrodynamic lift forces, which are more pronounced for the denser star polymers compared to the chains. To test this hypothesis, we conducted additional simulations where we switched off HI by employing a Langevin thermostat. At rest, the polymer distribution $P_{\mathrm{cm}}\left(x\right)$ looks identical to the data shown in Figure 2, as expected, since hydrodynamics do not affect the static properties at equilibrium. Flow was then applied to the system by superimposing a velocity profile with the same parabolic shape and amplitude as in our previous explicit solvent simulations. Here we found that the polymer distribution $P_{\mathrm{cm}}\left(x\right)$ was identical for all values of Rep, i.e., no cross-stream migration occurred in the simulations without hydrodynamics. This behavior can be rationalized by considering that the interaction matrix, relating the forces acting on the beads and their velocities, is diagonal when hydrodynamics are neglected; in this scenario, the motion along the individual directions (*x*, *y* and *z*) is fully decoupled and the underlying equations of motion can be solved independently. Hence, we can conclude that the cross-stream migration displayed in Figure 3 originates from hydrodynamic lift forces.

### 3.2. Polymer Mixtures

Our simulation results under ultradilute conditions revealed that the cross-stream migration behavior of polymers depends on their number of arms *f* (and thus their deformability), where polymers moved more and more towards the channel center with increasing *f*. This finding suggests the possibility of separating mixtures of polymers with different *f* *via* Poiseuille flow. To test this idea, we first prepared mixtures containing $N_{2}=13$ chains (f=2) and $N_{30}=13$ stars (f=30). This choice leads to a volume fraction of $\Phi =4\pi R_{g}^{3}\left(N_{2}+N_{30}\right)/\left(3V\right)\approx 0.1$, i.e., the system is still in the dilute regime (note that $R_{g}\approx 4.2$ for all investigated values of *f*).

**Figure 6:**
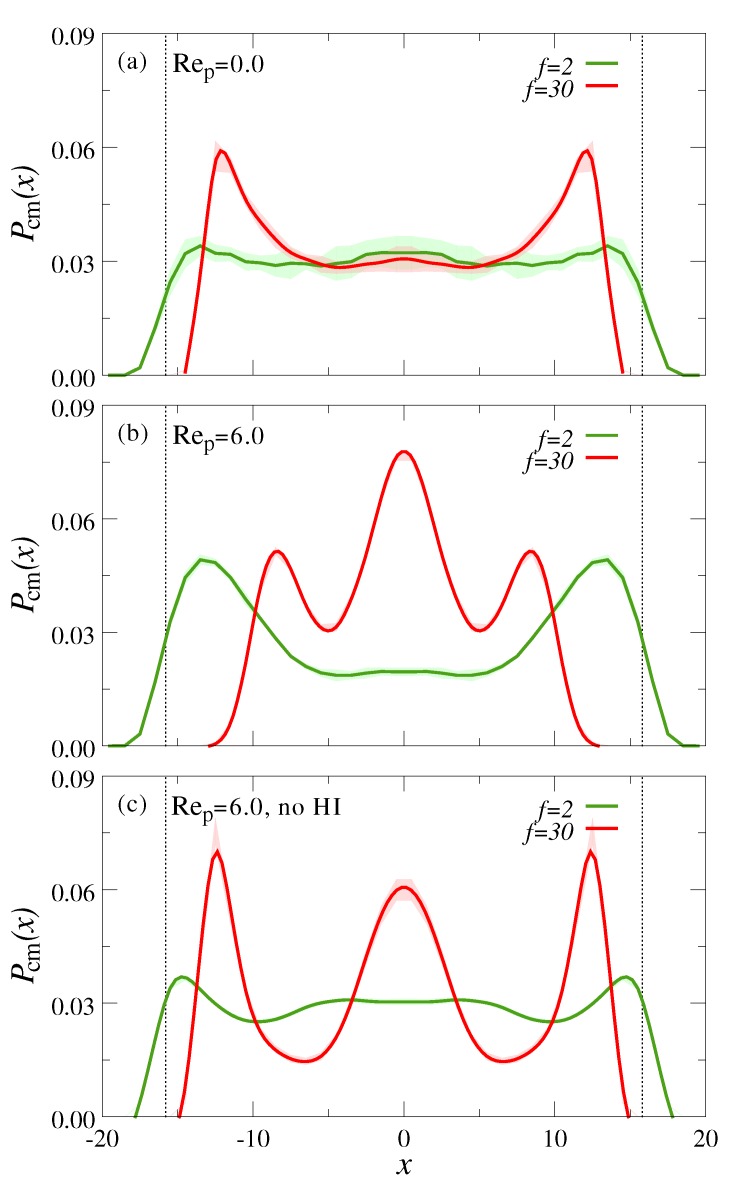
Center of mass probability distribution normal to the channel walls, $P_{\mathrm{cm}}\left(x\right)$, for a mixture of chains (f=2) and stars (f=30) at (**a**) rest (Rep=0) and (**b**) under flow (Rep=6). Panel (**c**) shows the system under flow (Rep=6), but with hydrodynamic interactions switched off. The volume fraction of polymers is fixed to Φ=0.1 in all simulations.

Figure 6 shows the probability distribution of the polymer CM between the channel walls, $P_{\mathrm{cm}}\left(x\right)$. At rest, the chains are uniformly distributed across the channel, similar to the case at infinite dilution (*cf.* Figure 2a). The stars, however, are not anymore uniformly distributed, but exhibit a distinct layering near the walls. This ordering is due to the fact that the effective interactions between star polymers become progressively hard-sphere like with increasing *f* [67,68]; for such (almost) hard spheres, ordered structures near a flat wall result from the excluded volume interactions both within the particles and against the confining wall [77,78,79,80].

When flow is applied, the star polymers vacate the region near the walls and migrate to the channel center, as evidenced by the distinct peak at x=0 shown in Figure 6b. We can identify two additional, slightly smaller peaks near the centerline at $x\approx \pm 2R_{g}$, which stem from the saturation of the centerline. At the same time, the linear polymers are largely expelled from the channel center and fill the region near the walls, where they attain a rather stretched out conformation. In principle, this clear spatial separation of chains and stars under flow allows for a straight-forward separation of the two species.

To elucidate the origin of this flow-induced separation, we again conducted simulations without HI. At rest, we find the exact same distribution as shown in Figure 6a, as expected. When flow is applied, we observe a qualitatively similar partial focusing of star polymers to the centerline with some differences (*cf.* Figure 6b,c): when HI are switched off, the chain distribution is somewhat more homogeneous and the two off-center peaks in the star distribution move now closer to the walls. The fact that flow influences the lateral distribution of polymers in the mixtures without HI (in contrast to the infinitely dilute systems discussed in Section 3.1) provides crucial insights to the responsible separation mechanism: the non-uniform shear field $\dot{\gamma }\left(x\right)$ leads to a more pronounced stretching of the chains near the walls (see Figure 4b), which thereby push the less deformable stars to the channel center. This effect is somewhat more pronounced in the simulations with HI, likely due to wall-induced hydrodynamic lift forces. We note that this behavior is reminiscent of the viscoelastic focusing of rigid colloids in polymer solutions [7,60,61]. However, the mass fraction of linear polymer in the present simulations is somewhat smaller (0.65%) than the typical values of 1–10% used in previous simulations [60,61] and experiments [6,7], which might explain the less pronounced focusing observed here.

**Figure 7:**
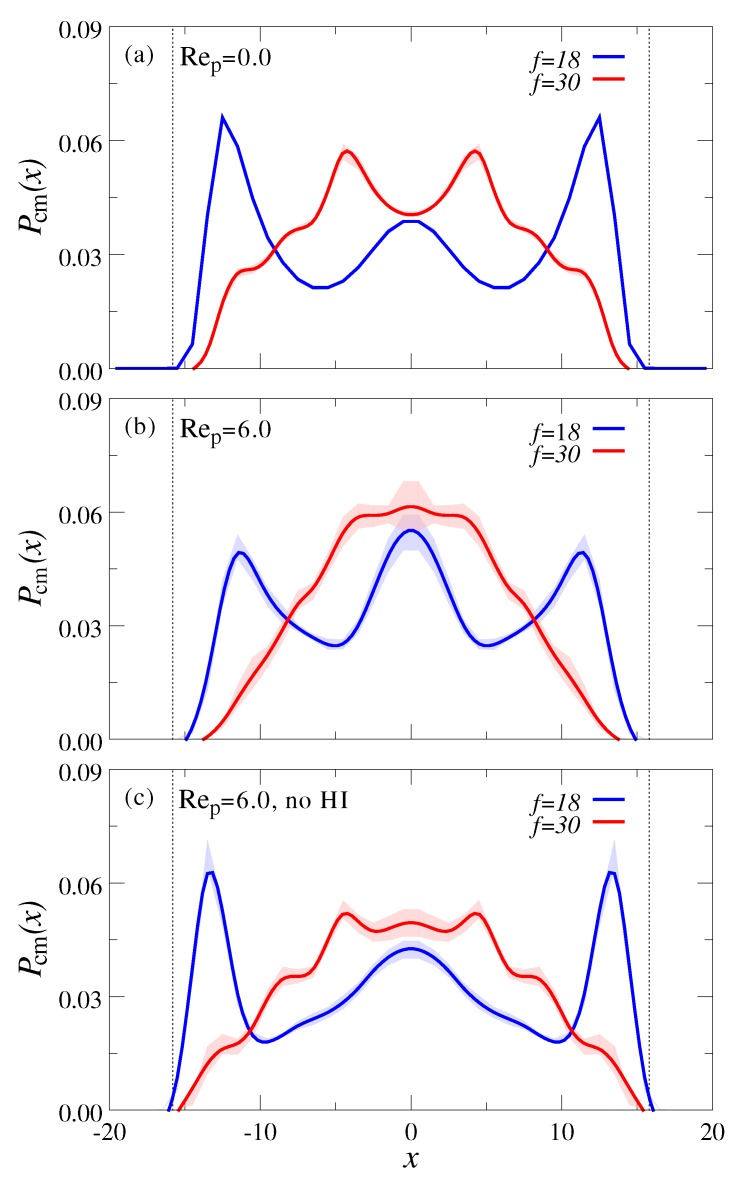
Center of mass probability distribution normal to the channel walls, $P_{\mathrm{cm}}\left(x\right)$, for a mixture of stars with f=18 and f=30 arms at (**a**) rest (Rep=0) and (**b**) under flow (Rep=6). Panel (**c**) shows the system under flow (Rep=6), but with hydrodynamic interactions switched off. The volume fraction of polymers is fixed to Φ=0.1 in all simulations.

To explore whether this setup can also be used to separate stars with different numbers of arms, we repeated our simulations for a mixture of $N_{18}=13$ and $N_{30}=13$ stars with f=18 and f=30 arms, respectively. The volume fraction is again Φ≈0.1. Figure 7a shows the equilibrium distribution of the stars between the walls, $P_{\mathrm{cm}}\left(x\right)$, and we can see again that the less deformable species (f=30) occupies the central channel region, whereas the softer particles (f=18) are pushed closer to the walls. Here, we can identify a distinct layering of the stars, which is more pronounced compared to the star-chain mixtures due to stronger excluded volume effects.

Under Poiseuille flow, stars with f=18 as well as f=30 arms move somewhat closer to the channel center, and the lateral layering is slightly smeared out (see Figure 7b). However, in contrast to the star-chain mixtures, there is no obvious spatial partitioning between the different star species which could be exploited for particle separation. When hydrodynamics are switched off, the lateral polymer distribution, $P_{\mathrm{cm}}\left(x\right)$, becomes slightly broader due to the lack of wall-induced hydrodynamic lift forces, but the overall behavior is qualitatively similar. The less pronounced flow-induced focusing in the star-star mixtures can thus be attributed to the weaker viscoelastic forces exerted by the stars compared to the chains.

For achieving a connection between our simulations and experiments beyond a comparison of dimensionless quantities (such as the Reynolds and Weissenberg number), it is required to establish a mapping between the units of energy, length and time. For the energy scale, we chose the thermal energy $\varepsilon =k_{B}T$, which at room temperature ($T_{\mathrm{room}}=298\;K$) is $\varepsilon =4.11\times 10^{-21}\;J$. For the length scale, we map our linear polymers to poly(ethylene oxide) (PEO) chains with molecular weight M=4000kg/mol, as used in previous viscoelastic focusing experiments [7]. The radius of gyration of PEO chains in water can be estimated *via* $R_{g}=0.215M^{0.583}$, with molecular weight *M* given in g/mol [81]. Thus, $R_{g}\approx 150\;\mathrm{nm}$ which leads to a conversion factor of σ≈36nm. (Please note that this mapping is rather crude, since roughly 2270 monomers are represented by a single bead, and thus the employed WCA excluded volume interactions between beads are likely too hard.) For the time scale, we matched the long-time diffusion coefficient *D* of a single chain at dilute conditions. In the simulations we find $D\approx 0.0045\sigma^{2}/\tau_{\mathrm{MD}}$ [38]. The diffusion coefficient of the experimental system can be estimated *via* $D=k_{B}T/\left(6\pi \eta_{s}R_{h}\right)$ with hydrodynamic radius $R_{h}$. Using $\eta_{s}=0.89\;\mathrm{cP}$ for water at room temperature and $R_{h}\approx 85\;\mathrm{nm}$ [81], we find $D\approx 2.9\times 10^{-8}\;{\mathrm{cm}}^{2}/s$ and thus $\tau_{\mathrm{MD}}\approx 2.0\;\mu s$. Using these conversion factors, our channels have a width of L≈1.5μm and the maximum fluid velocity is $v_{\max}\approx 1\;\mathrm{cm}/s$ at Rep=6. Such velocities can be achieved in a slit channel by applying a pressure drop of 100kPa over a channel length of 3mm. Flow-induced partitioning occurred in the star-chain mixtures within the simulation time (approximately 0.2s), which corresponds to a traveled distance of roughly 2mm at the highest employed flow strength. This distance is smaller than the channel length, and thus we expect that flow-induced separation of star and chain polymers should in principle be possible in experiments.

## 4. Conclusions

We performed explicit solvent molecular dynamics simulations of single chains, stars, and their mixtures under Poiseuille flow, and explored their conformation and cross-stream migration. We found that at infinite dilution, star polymer experienced stronger lift forces to the channel center compared to their linear counterparts with the same equilibrium radius of gyration. By conducting additional simulations without hydrodynamics, we identified wall-induced hydrodynamic lift forces as the mechanism responsible for this lateral motion.

In the star-chain mixtures, we observed an even more pronounced spatial separation of the two species, where the stars occupied the central channel region and the more deformable chains moved closer to the channel walls. In contrast, the flow-induced demixing of star-star mixtures was much less pronounced. In principle, such flow-induced partitioning can be exploited to filter polymers based on their architecture. Our simulations indicate that the spatial separation in the polymer mixtures stems from a combination of wall-induced hydrodynamic lift forces and viscoelastic forces originating from the linear chains. Hence, the phenomena observed here appear to be more closely related to the viscoelastic focusing of (rigid) colloids than to the deformation-induced lift of elastic capsules.

## Abbreviations

The following abbreviations are used in this manuscript:

CM	Center of mass
FENE	Finitely extensible nonlinear elastic
HI	Hydrodynamic interactions
MD	Molecular Dynamics
MPCD	Multi-particle collision dynamics
PDMS	Polydimethylsiloxane
PEO	Poly(ethylene oxide)
WCA	Weeks-Chandler-Andersen
